# COVoc and COVTriage: novel resources to support literature triage

**DOI:** 10.1093/bioinformatics/btac800

**Published:** 2022-12-13

**Authors:** Déborah Caucheteur, Zoë May Pendlington, Paola Roncaglia, Julien Gobeill, Luc Mottin, Nicolas Matentzoglu, Donat Agosti, David Osumi-Sutherland, Helen Parkinson, Patrick Ruch

**Affiliations:** SIB Text Mining Group, Swiss Institute of Bioinformatics, Geneva 1206, Switzerland; BiTeM Group, Information Sciences, HES-SO/HEG Genève, Carouge 1227, Switzerland; European Molecular Biology Laboratory, European Bioinformatics Institute (EMBL-EBI), Wellcome Genome Campus, Cambridge CB10 1SD, UK; European Molecular Biology Laboratory, European Bioinformatics Institute (EMBL-EBI), Wellcome Genome Campus, Cambridge CB10 1SD, UK; SIB Text Mining Group, Swiss Institute of Bioinformatics, Geneva 1206, Switzerland; BiTeM Group, Information Sciences, HES-SO/HEG Genève, Carouge 1227, Switzerland; SIB Text Mining Group, Swiss Institute of Bioinformatics, Geneva 1206, Switzerland; BiTeM Group, Information Sciences, HES-SO/HEG Genève, Carouge 1227, Switzerland; Department of Microbiology and Molecular Medicine, Faculty of Medicine, University of Geneva, Geneva 1205, Switzerland; European Molecular Biology Laboratory, European Bioinformatics Institute (EMBL-EBI), Wellcome Genome Campus, Cambridge CB10 1SD, UK; Semanticly Ltd, London, WC2H 9JQ, UK; SIB Text Mining Group, Swiss Institute of Bioinformatics, Geneva 1206, Switzerland; Plazi, Bern 3007, Switzerland; European Molecular Biology Laboratory, European Bioinformatics Institute (EMBL-EBI), Wellcome Genome Campus, Cambridge CB10 1SD, UK; European Molecular Biology Laboratory, European Bioinformatics Institute (EMBL-EBI), Wellcome Genome Campus, Cambridge CB10 1SD, UK; SIB Text Mining Group, Swiss Institute of Bioinformatics, Geneva 1206, Switzerland; BiTeM Group, Information Sciences, HES-SO/HEG Genève, Carouge 1227, Switzerland

## Abstract

**Motivation:**

Since early 2020, the coronavirus disease 2019 (COVID-19) pandemic has confronted the biomedical community with an unprecedented challenge. The rapid spread of COVID-19 and ease of transmission seen worldwide is due to increased population flow and international trade. Front-line medical care, treatment research and vaccine development also require rapid and informative interpretation of the literature and COVID-19 data produced around the world, with 177 500 papers published between January 2020 and November 2021, i.e. almost 8500 papers per month. To extract knowledge and enable interoperability across resources, we developed the COVID-19 Vocabulary (COVoc), an application ontology related to the research on this pandemic. The main objective of COVoc development was to enable seamless navigation from biomedical literature to core databases and tools of ELIXIR, a European-wide intergovernmental organization for life sciences.

**Results:**

This collaborative work provided data integration into SIB Literature services, an application ontology (COVoc) and a triage service named COVTriage and based on annotation processing to search for COVID-related information across pre-defined aspects with daily updates. Thanks to its interoperability potential, COVoc lends itself to wider applications, hopefully through further connections with other novel COVID-19 ontologies as has been established with Coronavirus Infectious Disease Ontology.

**Availability and implementation:**

The data at https://github.com/EBISPOT/covoc and the service at https://candy.hesge.ch/COVTriage.

## 1 Introduction

At the end of 2019, a severe form of viral pneumonia was detected in China. The majority of the initial cases had visited a local market where the sale of live animals was allowed. However, it was subsequently found that people who had not visited the market were also carrying the virus, concluding that human–human transmission was possible. The first death officially reported by the authorities occurred on January 11, 2020 (https://www.who.int/csr/don/12-january-2020-novel-coronavirus-china/en/). The flow of people and merchandise led to a rapid spread of the virus around the globe, sparing no continent. Although many measures (incl. barrier gestures, curfews and confinement) may have slowed the spread of the disease, the threshold of more than 1 million reported deaths was exceeded during the year 2020.

The prompt response of the scientific community to the pandemic led to the publication of a large number of scientific articles (Chen *et al.*, 2020) including preprints. Nevertheless, it is not always easy for a researcher to find information that is relevant to them in the midst of this large collection. Coronavirus disease 2019 (COVID-19) itself was only defined as ‘aggravated pneumonia’ at the beginning of the pandemic and the virus as ‘novel coronavirus’, but no official name or definition was declared until early 2020. In January 2020 (https://www.who.int/docs/default-source/coronaviruse/situation-reports/20200130-sitrep-10-ncov.pdf), ‘2019 n-CoV’ was the first name proposed by the WHO before the final decision was declared in February: ‘COVID-19’ for ‘coronavirus disease 2019’ (https://www.who.int/docs/default-source/coronaviruse/situation-reports/20200211-sitrep-22-ncov.pdf).

This new disease has led to the use of new terms, making the retrieval of information more complex. An ontology dedicated to COVID-19 would be very useful to achieve efficient and quick queries by scientific researchers. By definition, an ontology is a controlled vocabulary that structures defined terms with the use of hierarchical relationships and is a mathematical model based on a subset of first-order logic. Ontologies additionally contain synonyms and cross-references to other ontologies, vocabularies and other resources to enrich each term and improve the harmonization and interoperability of annotated data and literature. As a result, ontologies are both human- and computer-readable and can aid in analysis. There are two main types of ontologies: domain and application. A domain ontology consists of knowledge centered on a specific entity or entities within the same scope (e.g. cells, anatomical structures or phenotypes) and the relations between them. Many domain ontologies in the biomedical space abide by the standards set out by and are part of the Open Biological and Biomedical Ontologies (OBO) Foundry (Jackson *et al.*, 2021). An application ontology can be built to define a wider set of entities than a domain ontology, for example an ensemble of cells, anatomical structures, phenotypes, diseases and assays can be combined in an application ontology to detail the interactions and relationships between the individual domains while using the expertly curated terms and relationships from the domain ontologies. Domain ontologies are regularly created and driven by data or application needs, as an alternative to duplicating work and creating new domain ontologies. The benefit of an application ontology comes from relating domain-specific ontology terms to each other, allowing new relationships to be visualized (e.g. cells linked to anatomy, anatomy linked to phenotypes and phenotypes linked to disease).

To serve scientific research, data sharing and information dissemination, we worked in collaboration with committed research partners to develop:

- a controlled vocabulary dedicated to COVID-19 named COVoc,
- an application ontology from the controlled vocabulary,
- associated text analytics services (e.g. COVTriage).

We participated in the TREC-COVID competition (https://ir.nist.gov/covidSubmit/index.html) to assess the performance of these tools.

Established in 1992, the Text REtrieval Conference (TREC) (https://trec.nist.gov), co-sponsored by the United States National Institute of Standards and Technology (NIST) and the Intelligence Advanced Research Projects Activity, is a yearly workshop focused on a list of different information retrieval (IR) research areas, also named tracks. The goal is to accelerate research in this domain. With the creation of the first large test collections of full-text documents and standardized retrieval evaluation and a consequent number of participants and edition, the TREC competition has contributed to the development of new retrieval techniques.

The BiTeM group at HES-SO and SIB Text Mining group at the Swiss Institute of Bioinformatics (SIB) in Geneva have been participating in several TREC tracks like TREC Clinical Decision Support (Gobeill *et al.*, 2014, 2015), TREC Chemical IR (Gobeill *et al.*, 2011a), TREC Genomics (Gobeill *et al.*, 2007), TREC Medical Records (Gobeill *et al.*, 2011b), TREC Deep Learning (Knafou *et al.*, 2019, 2020) and TREC Precision Medicine (Caucheteur *et al.*, 2019; Pasche *et al.*, 2017, 2018, 2020).

On April 15th, 2020, NIST announced the launch of TREC-COVID, a community evaluation that created a collection of tests around the literature specific to COVID-19, provided by the Allen Institute for Artificial Intelligence (AI2). In the context of the pandemic, the topics of interest, number of publications and diversity of the covered subjects are changing rapidly. To remain effective in supporting biomedical research for information retrieval (in the context of the TREC competition for example), it is necessary to provide useful data for the evaluation of algorithms or to discover and manage scientific information methods for future biomedical crises.

## 2 Materials and methods

### 2.1 COVoc—ontology development

In March 2020, we started collaborating on a public spreadsheet with a wide range of research communities to collect vocabulary from scientific publications or press articles related to the COVID-19 pandemic. To this aim, several hackathons were held. In addition, a list of terms was automatically generated containing the most frequent terms used in abstracts of the CORD-19 collection (Wang *et al.*, 2020), a collection dedicated to COVID-19 research provided by the AllenAI Institute, according to a TF-IDF (term frequency-inverse document frequency) calculation script. The top vocabulary terms were manually curated and added to a spreadsheet in order to organize the axes according to individual concepts and to allow an easy, user-friendly way for collaborative contributions from the scientific community without the requirement for ontology knowledge.

An ontology-building pipeline was designed to compile the resource directly from the spreadsheet to create an application ontology with the distinct purpose of aiding the curation of COVID-19-related literature. Classes from existing OBO ontologies were imported where applicable, along with their cross-references to public resources, making COVoc highly interoperable with existing domain ontologies and the growing community of COVID-related ontologies in response to the pandemic. Manual and automated curation was carried out to create the template files from the original spreadsheet, using available mapping tools Zooma (http://wwwdev.ebi.ac.uk/spot/zooma/) and the Ontology Lookup Service (https://www.ebi.ac.uk/ols/index). The objective was to include as many terms as possible from widely used OBO ontologies to connect annotations to COVoc directly to other useful resources such as the COVID-19 data platform (https://www.covid19dataportal.org/) (Table 1) and utilize domain ontology expertise alongside minting novel COVID-19 related terms. Terms that are both novel to COVoc and imported from other ontologies have additional annotation properties to aid users of COVoc, including a preferred label, synonyms, internal identifiers, cross-references to both OBO ontologies and other vocabularies and resources. The Ontology Development Kit (https://github.com/INCATools/ontology-development-kit) and ROBOT (http://robot.obolibrary.org/) were used to convert the templates from the original spreadsheet. Protégé (version 5.5.0) (https://protege.stanford.edu) was used to check the structure of COVoc during the ontology-building process.

**Table 1.** Mapping among 20 existing ontologies for each COVoc axis

Axis	Cross-references with:
BioMedical Vocabulary	BAO, CHEBI, CHIMO, CL, EFO, GO, IDO, MAXO, Mondo, NCBITaxon, NCIt, OBI, OMT, PR and UBERON
Biotic interactions	ENVO, GO, INO, NBO, OMIT and RO
Cell lines	CLO and EFO
Chemicals	CHEBI and NCIt
Clinical trials	—
Conceptual entities	CHEBI, EFO, OBI and PATO
Diseases and syndromes	CHEBI, EFO, HP and Mondo
Geographic locations	DBPedia and HANCESTRO
Organisms	NCBITaxon and CIDO
Proteins and genomes	CHEBI, NCIt and PR

### 2.2 Integration into SIBiLS

The first step is the import of literature collections into SIBiLS (Swiss Institute of Bioinformatics Literature Services) (Gobeill et al., 2020). We work with three collections: MEDLINE (https://www.nlm.nih.gov/bsd/medline.html), free full-text from PMC (https://europepmc.org/About) and CORD-19 (Wang *et al.*, 2020), respectively consisting of 34 664 562, 4 722 601 and 389 830 documents (October 2022). These collections are daily updated for the first two, punctually for the third. Additional collections regrouping supplementary data files from PMC as well as taxonomic treatments from Plazi (Naderi et al., 2022; Penev et al., 2022) are planned.

A parsing task is done to split each document into distinct fields, for example title, abstract, keywords, which are then pushed into a MongoDB database.

Document annotation is a once-only process. First, it implies string pre-processing and tokenization methods. A dash or a slash could sometimes be responsible for non-matching. In our pipeline, this risk is removed thanks to the deletion of these symbols and the creation of additional words: the two parts of words are kept separately but also fused to create a new word (‘covid-19’ becomes ‘covid’ plus ‘covid19’, while ‘19’ is not kept because its length is inferior to three characters). Such processing enables retrieval of papers in which only occurrences of the word with the dash are present for example. Applied to documents as well as to ontology concepts, this processing set makes it possible to annotate terms no matter how the author has spelled them. The second step is the use of COVoc ontology to annotate the collections: identifiers of COVoc concepts found in the text are attached to it. By going through each document in a corpus, we question its presence in one of the COVoc axes. When a term is matched, an annotation is constructed which includes the term, its id, the associated preferred term, the axis in which it is found and its position in the document. To access these COVoc annotations in a dedicated way, we have developed the COVTriage tool (previously named COVID Triage) described in the next section. To allow a filtering step by users, COVoc is split into axes: BioMedical Vocabulary (BMV), Cell lines (CL), Clinical Trials (CT), Conceptual entities (CE), Diseases and Syndromes (DIS), Chemicals (CHEM), Geographic locations (GL), Organisms (ORG) and Proteins and Genomes (PG). If users are more interested in the cell lines cited in publications, it will be possible to show only the ‘Cell lines’ axis annotations.

The benefits of this pipeline are multiple:

- time saving when answering queries because even in the case of repeated queries, the process has been carried out beforehand, only once. Also, paragraphs are reduced to a list of IDs where one or more IDs are retrieved, which is faster;
- a better recall thanks to the association of a unique identifier for each occurrence of a concept and all its synonyms.

For each document loaded in the initial MongoDB database, a list of related annotations is created and exported as a unique entry in a new MongoDB database, dedicated to annotations. Finally, both original fields and annotations are indexed in an ElasticSearch index (v7.2.0).

### 2.3 CoVTriage—service

COVTriage is a prioritization system designed to facilitate the literature investigation process for clinicians and researchers involved in the fight against COVID-19.

Initially built as a prototype to demonstrate the typical use case of the COVoc ontology, we have then decided to carry on the development and the maintenance of this application that responds concretely to this research needs. COVTriage aims at providing the experts with relevance-based articles from three different collections: MEDLINE, PubMed Central (PMC) and COVID-19 Open Research Dataset (CORD-19) (Wang *et al.*, 2020).

Developed with Python/Java/Javascript technologies, COVTriage implements a search engine based on the SIB Literature Services (SIBiLS) (Gobeill et al., 2020), a graphical user interface (GUI) and a bunch of APIs enabling interactions with other systems.

On the back end of COVTriage, MongoDB hosts a mirror of the literature and the full set of annotations resulting from the above-mentioned work with COVoc. Different ElasticSearch indexes provide quick access to these elements depending on the source selected at query time by the user. Their request is processed on the server side and must include a ‘focus’ in addition to the keywords. This focus can be selected among the nine COVoc axes and it will impact the ranking of the retrieved documents. That is, for a specific search, a score is calculated for each returned article according to its proximity to the initial query and its relevance to the selected focus. Details about this re-ranking (RR) function and its evaluation are presented in Sections 2.4 and 3.3.

On the front end, a graphical user interface (GUI) has been implemented by adapting a layout that has already proven effective for biomedical literature curation (Britan *et al.*, 2018). As a complement to the website, public REST APIs were prepared to ensure the interoperability of the two major functionalities (literature prioritization and annotation extraction) with independent systems.

### 2.4 Evaluation (TREC-COVID)

The TREC-COVID task was a classic *ad hoc* search task: from a pandemic-related topic (i.e. query like ‘what evidence is there related to COVID-19 super spreaders’), the competing system had to return a ranked list of 1000 relevant documents. The document set used was the CORD-19 collection and the relevant judgements were made by human experts. As five different and successive rounds were organized, the collection and topic lists were updated for each round. For Round 3, the CORD-19 collection (May 19 version) contained 54 842 documents and there were 40 topics; for Round 4, the collection (June 19 version) contained 73 858 documents and there were 45 topics. TREC-COVID brought together hundreds of participants for five rounds of evaluation where each team could complete five runs, five lists of ranked documents per topic from distinct systems (https://ir.nist.gov/covidSubmit/papers/Forum_TRECCOVID1.pdf).

Our system relies on robust strategies, and strategies linked with the COVoc ontology. First, we parsed the collection and indexed documents and metadata in a Lucene Elasticsearch engine. For each topic, we generated a weighted set of keywords based on terms’ Document Frequencies observed in PubMed Central rather than in CORD-19; this strategy aimed at favoring rare and informative words. The engine was then queried with Okapi BM25 weighting scheme, in order to generate baseline runs. Beyond this, we also exploited the COVoc ontology for producing supplementary runs. The COVoc terms were mapped in the CORD-19 collection, and documents enriched with mapped COVoc concept ids were indexed in a second Elasticsearch index; in the same way, the mapping was applied to topics to expand the queries with relevant COVoc concept ids. The engine was then queried to generate the so-called ‘query expansion’ runs. Finally, the search engine implemented in COVTriage was applied to both baseline and query expansion runs.

To refine the process, we developed a prioritization component that takes further advantage from COVoc to contextualize the search. At least one of the ontology axes is *a priori* associated with each of the TREC queries with regards to their proximity. At query time, the system gathered the list of publications returned by the search engine together with all the concepts stored into SIBiLS for the selected axis. Then, the list is re-ranked according to a new score calculated with a linear combination of three parameters: (i) the original score from ElasticSearch; (ii) a measurement of the document specificity (the number of distinct concepts related to the selected axis); and (iii) a measurement of COVoc density in the document (the TF-IDF). The final linear combination has been optimized with weighting factors empirically set at Round 3 of TREC-COVID to generate re-ranking runs (RR) for the evaluation.

## 3 Results

### 3.1 Ontology

COVoc contains 563 terms with 2481 synonyms, required to enhance annotation and search functions and 5751 cross-references, enhancing the harmonization and interoperability of data annotated to COVoc.

Over 400 COVoc terms have been mapped to and imported from one of 20 existing ontologies (Table 1), mostly in the biological/medical vocabulary, diseases and proteins name spaces. Cell lines were enriched with synonyms from the CelloSaurus (Bairoch, 2018), which is now an ELIXIR Core Data Resource. Additional synonyms and cross-references added to these terms will be sent to the domain ontologies in question in order to improve the terms at their core. In early 2023, the biotic interaction descriptors benefited from the contribution of the biodiversity community via the CETAF/DISSCo Task Force (Poelen *et al.*, 2020) will be added to COVoc.

Around 100 novel COVoc terms were created for concepts that did not exist in other ontologies or for entirely new concepts that fit the COVoc ontology space (e.g. terms for clinical trials). These terms are currently under review to be included in CIDO (Coronavirus Infectious Disease Ontology (https://github.com/CIDO-ontology/cido; He *et al.*, 2020, 2022), Cell Ontology (CL; Diehl *et al.*, 2016) or other domain ontologies where possible.

The ontology can be browsed using the Ontology Lookup Service (OLS) (Jupp *et al.*, 2015) and is available with the full ontology building pipeline on GitHub (https://github.com/EBISPOT/covoc). The curation-support literature triage demonstrator is available online (https://candy.hesge.ch/COVTriage).

### 3.2 SIBiLS—annotation process

Those COVoc concepts and synonyms were used to fully annotate biomedical literature from MEDLINE, PubMed Central as well as the COVID-19 Open Research Dataset (CORD-19) preprint collection provided by the AllenAI institute as described before.

By October 2022, we had more than a billion COVoc annotations (*N* = 1 089 914 909) which can be accessed via SIBiLS (https://sibils.github.io).

Statistics have been calculated on our different collections including the number of annotations per axis per collection, the total number of annotations per collection and the relative average (Table 2). As expected, the average of annotations per document is higher for the CORD-19 collection (*n *= 219), because these documents are dedicated to COVID-19 like COVoc ontology. The CORD-19 annotations are uploaded within the EuropePMC archive using the SciLite services (Venkatesan *et al.*, 2017).

**Table 2.** Statistics for COVoc annotations into SIBiLS (October 2022)

COVoc axes	MEDLINE	ePMC	CORD-19
BMV	175 386 868	441 873 718	36 199 118
CE	53 959 676	150 125 656	19 585 865
CL	173 861	872 857	203 926
CT	0	3	1406
CHEM	2 194 487	5 253 987	676 882
DIS	27 828 170	67 832 153	11 350 128
GL	3 981 568	16 417 448	1 845 734
ORG	12 385 979	32 341 398	13 315 914
PG	3 624 982	10 261 638	2 221 487
Total annotations	279 535 591	724 978 858	85 400 460
No. of documents	34 664 562	4 722 601	389 830
Average anns/doc	8	153	219

### 3.3 Evaluation (TREC-COVID)

Metrics evaluation of our two strategies [query expansion (QE) and re-ranking (RR)] are available in Table 3. Experiments no. 1 and no. 2 correspond to Round 3 and Round 4 of TREC-COVID, respectively. As shown, the evolutionary rate of P@10 in Experiment no. 1 is positive (+19.7%) whereas for MAP it is quite stable (+3.97%). In Experiment no. 2, it is the evolutionary rate of P@10 which is rather stable (−2.88%) and that of MAP is strongly positive (+54.77%). COVoc, exploited via QE strategy and/or reranking strategy, has a statistically significant positive effect compared to the baseline.

**Table 3.** Metrics evaluations in Experiments no. 1 and no. 2

	P@10	Ev. rate	MAP	Ev. rate
Experiment no. 1				
Baseline	0.4825		0.1561	
+QE	0.555		0.158	
+RR	0.41		0.1436	
+QE +RR	0.575	+19.17%	0.1623	+3.97%
Experiment no. 2				
Baseline	0.6178		0.1006	
+QE	0.6022		0.1519	
+RR	0.6244		0.1708	
+QE+RR	0.6	−2.88%	0.1557	+54.77%

Ev. rate, evolutionary rate (compared to the baseline results).

### 3.4 CoVTriage

**Fig. 1.**
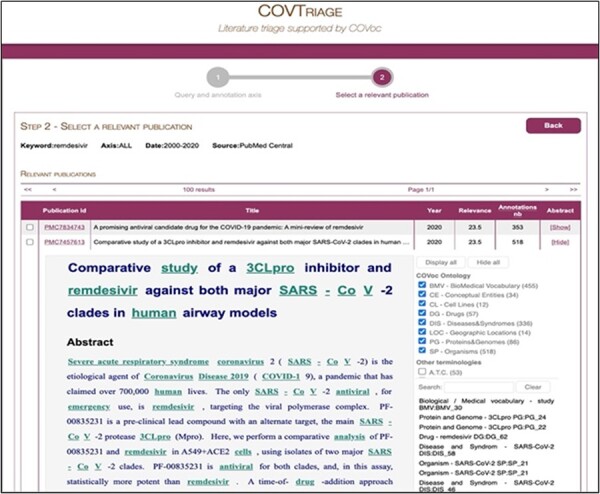
GUI of COVTriage. COVTriage display of search results for the query ‘remdesivir’. Articles are retrieved in PMC in the range 2000–2020

COVTriage is a web application publicly available since April 2020. Besides the mandatory input {keywords+focus} on the first panel, the user can fill in some optional fields such as the source (otherwise MEDLINE is selected by default), a date range related to the articles’ publication dates, or keywords to filter out some results. Then, once the documents have been processed, the ranked result of the retrieval function is displayed on the second panel. At this step, the user can browse through the articles of interest and take advantage of the highlighting of COVoc concepts as presented in Figure 1. Supplementary concepts from other biomedical ontologies (MeSH, ATC, ICD-10, …) can also be highlighted at this step if the user checks the corresponding boxes on the right frame. Finally, if needed, the user can export selected publications with their integrated annotations into a json output.

All the data generated and visualized in COVTriage may represent a substantial resource for research groups. Our tool accumulates around 1754 visits, with an average of 3.1 queries per day, from individuals around the world between June 2020 and December 2021.

In order to enable programmatic searches and to make the data directly integrable into external processing pipelines, we have developed public web services. Accessible through REST APIs, they ensure the interoperability with independent systems by providing json outputs for the two possible requirements: literature prioritization and annotation extraction. Details on how to use the services are described in the documentation [https://sibils.github.io; https://candy.hesge.ch/COVTriage/documentation/).

## 4 Discussion

COVoc, via COVTriage which provides a dimension-specific prioritization of the literature based on the context of documents, is a text-mining tool for research scientists looking for publications about precise axes around the COVID-19 pandemic. Nevertheless, at least one axis needs to be improved. As observed in the statistics table, there is almost no annotation for the Clinical Trials (CT) axis, which means no NCTid (National Clinical Trial identifier) is matched. More work needs to be done to check why they are not detected by our system. To be more efficient in the detection of Clinical Trials through publications, it could be useful to add specific terms related to Clinical trials in the CT axis. This should reveal the papers where a clinical trial is discussed but not systematically point to the clinical trial ID linked. Another approach could be to look for the PMID or PMCID of each paper from the CORD-19 collection in the Clinical Trials collection available in SIBiLS, specifically based on the ‘PMID (PMCID) reference’ field available from CT.gov to retrieve clinical trials linked (with the NCTid) to these publications and create in a second time the corresponding annotation. Furthermore, with the research on COVID-19, the number of clinical trials and their citations have risen significantly (around 4000 in December 2020, 7154 1 year later) (https://clinicaltrials.gov/ct2/results?cond=COVID-19). With the COVID-19 vaccine campaigns, the Clinical Trials axis should prove to be more consistent, and the number of annotations should increase.

COVoc annotations are also available to the scientific community via EuropePMC. To have an overview of the integration of these annotations, by executing the query [(ANNOTATION_TYPE:‘COVoc’) AND (ANNOTATION_PROVIDER:‘HES-SO_SIB’)], all papers with at least one COVoc annotation will be retrieved.

During 2021, we engaged in an effort to harmonize COVID-19 ontologies created in response to the pandemic, contributing to the 12th International Conference on Biomedical Ontologies (ICBO 2021) (https://icbo2021.inf.unibz.it/) flash talk ‘*A community effort for COVID-19 Ontology Harmonization*’. Through this, we began collaboration efforts with CIDO (He *et al.*, 2020, 2022), a COVID-19 domain ontology which partly overlaps with COVoc. As COVoc is an application ontology, it is more ideal to import from domain ontologies which have been developed and curated by field experts and will continue to be updated to contain the latest information. This will enhance COVoc in the long term as we will benefit from expert curation and the continuous updates through dynamic import of domain ontologies like CIDO, Mondo and the Cell Line Ontology (CLO) in response to the COVID-19 pandemic and continuing research. This is an ongoing project where we hope to reduce the number of terms minted in the COVoc namespace and instead import from domain ontologies where possible and improve the annotation of unique COVoc terms that do not have an exact domain ontology such as the clinical trial terms.

In addition to financial resources, it is worth observing that the long-term maintenance of COVTriage may be affected by copyright regulations. Indeed, the pandemics imposed Open Access as the de facto standard for scientific publications and although Open Access is benefiting from a clear impetus, many Open Access contents may return to a paywalled status once the pandemic is declared over. Further, the WHO-promoted ‘One Health’ paradigm needed to respond to the health crisis and in particular zoonosis do demand for more holistic/inclusive perspectives over life sciences libraries. The current topical boundaries of MEDLINE and PMC, which excludes relevant contributions from non-medical sciences, and in particular biodiversity, should opportunely be questioned. These questions emerged on the agenda of both SIB and ELIXIR and will require innovative responses at a global level with potential implications from leading publishers in the field. The BICIKL project, coordinated by Pensoft, could play a pioneering role with the development of a Biodiversity Knowledge Hub (Penev *et al.*, 2022), i.e. a one-stop entry point to search publications and data from all life sciences.

## 5 Conclusion

Thanks to the work of collaborators since the first months of the pandemic, a controlled vocabulary about COVID-19 was developed to help scientific communities. COVoc is now translated into application ontology to meet needs such as interoperability, and totally dedicated to COVID-19. The first release is available on GitHub (https://github.com/EBISPOT/covoc) and ontology comments and requests can be submitted via https://github.com/EBISPOT/covoc/issues. It is also used as a controlled vocabulary for the triage tool named COVTriage (https://candy.hesge.ch/COVTriage). The literature ranking service in response to a query is based on COVoc concepts found in text corpora and created in the form of annotations. This helps researchers to deal with the large amount of new information about COVID-19. These annotations are uploaded and available via the Europe PMC website (https://europepmc.org), directly in the publication viewer interface. The presentation of COVoc at congresses has aroused interest and should lead to new collaborations in the near future. A first collaboration is already underway with CIDO.

## References

[btac800-B1] Bairoch A. (2018) The cellosaurus, a cell-line knowledge resource. J. Biomol. Tech., 29, 25–38. 29805321 10.7171/jbt.18-2902-002 PMC5945021

[btac800-B2] Britan A. et al (2018) Accelerating annotation of articles via automated approaches: evaluation of the neXtA5 curation-support tool by neXtProt. Database, 2018. 10.1093/database/bay129. PMC6301339 30576492

[btac800-B3] Caucheteur D. et al (2019) Designing retrieval models to contrast precision-driven ad hoc search vs. recall-driven treatment extraction in precision medicine. In: *Text REtrieval Conference (TREC), 2019, NIST (National Institute of Standards and Technology)*, Gaithersburg, Maryland.

[btac800-B4] Chen Q. et al (2020) LitCovid: an open database of COVID-19 literature. Nucleic Acids Res., **49**(D1), D1534–D1540. 10.1093/nar/gkaa952. PMC7778958 33166392

[btac800-B5] Diehl A. et al (2016) The cell ontology 2016: enhanced content, modularization, and ontology interoperability. J. Biomed. Semantics, 7, 44. 27377652 10.1186/s13326-016-0088-7 PMC4932724

[btac800-B6] Gobeill J. et al (2007) Vocabulary-driven passage retrieval for question-answering in genomics. In: *Text REtrieval Conference (TREC), 2007, NIST (National Institute of Standards and Technology)*, Gaithersburg, Maryland.

[btac800-B7] Gobeill J. et al (2011a) BiTeM group report for TREC chemical IR track 2011. In: *Text REtrieval Conference (TREC), 2011, NIST (National Institute of Standards and Technology)*, Gaithersburg, Maryland.

[btac800-B8] Gobeill J. et al (2011b) BiTeM group report for TREC medical records track 2011. In: *Text REtrieval Conference (TREC), 2011, NIST (National Institute of Standards and Technology)*, Gaithersburg, Maryland.

[btac800-B9] Gobeill J. et al (2014) Full-texts representation with medical subject headings and co-citations network reranking strategies for TREC 2014 clinical decision support track. In: *Text REtrieval Conference (TREC), 2014, NIST (National Institute of Standards and Technology)*, Gaithersburg, Maryland.

[btac800-B10] Gobeill J. et al (2015) Exploiting incoming and outgoing citations for improving information retrieval in the TREC 2015 clinical decision support track. In: *Text REtrieval Conference (TREC), 2015, NIST (National Institute of Standards and Technology)*, Gaithersburg, Maryland.

[btac800-B11] Gobeill J. et al (2020) SIB literature services: RESTful customizable search engines in biomedical literature, enriched with automatically mapped biomedical concepts. Nucleic Acids Res., 48, W12–W16. 32379317 10.1093/nar/gkaa328 PMC7319474

[btac800-B12] He Y. et al (2020) CIDO, a community-based ontology for coronavirus disease knowledge and data integration, sharing, and analysis. Sci. Data, 7, 181. 32533075 10.1038/s41597-020-0523-6 PMC7293349

[btac800-B13] He Y. et al (2022) A comprehensive update on CIDO: the community a-based coronavirus infectious disease ontology. J. Biomed. Semantics, 13, 25. 36271389 10.1186/s13326-022-00279-z PMC9585694

[btac800-B14] Jackson R. et al (2021) OBO Foundry in 2021: operationalizing open data principles to evaluate ontologies. Database, 2021. 10.1093/database/baab069 PMC8546234 34697637

[btac800-B15] Jupp S. et al (2015) A new Ontology Lookup Service at EMBL-EBI. In: Malone J (ed.) *Proceedings of SWAT4LS International Conference 2015*, Cambridge, UK.

[btac800-B16] Knafou J. et al (2019) SIB text mining at TREC 2019 deep learning track: working note. In: *Text REtrieval Conference (TREC), 2019, NIST (National Institute of Standards and Technology)*, Gaithersburg, Maryland.

[btac800-B17] Knafou J. et al (2020) SIB text mining at TREC 2020 deep learning track. In: *Text REtrieval Conference (TREC), 2020, NIST (National Institute of Standards and Technology)*, Gaithersburg, Maryland.

[btac800-B18] Naderi N. et al (2022) Analyzing the information content of text-based files in supplementary materials of biomedical literature. Stud. Health Technol. Inform., 294, 876–877. 35612233 10.3233/SHTI220614

[btac800-B19] Pasche E. et al (2017) Customizing a variant annotation-support tool: an inquiry into probability ranking principles for TREC precision medicine. In: *Text REtrieval Conference (TREC), 2017, NIST (National Institute of Standards and Technology)*, Gaithersburg, Maryland.

[btac800-B20] Pasche E. et al (2018) SIB text mining at TREC 2018 precision medicine track. In: *Text REtrieval Conference (TREC), 2018, NIST (National Institute of Standards and Technology)*, Gaithersburg, Maryland.

[btac800-B21] Pasche E. et al (2020) SIB text mining at TREC precision medicine 2020. In: *Text REtrieval Conference (TREC), 2020, NIST (National Institute of Standards and Technology)*, Gaithersburg, Maryland.

[btac800-B22] Penev L. et al (2022) Biodiversity community integrated knowledge library (BiCIKL). Res. Ideas Outcomes, 8, e81136.

[btac800-B23] Poelen J. et al (2020) CETAF-DiSSCo/COVID19-TAF biodiversity-related knowledge hub working group: indexed biotic interactions and review summary (0.2). [Data set]. Zenodo. 10.5281/zenodo.3839098.

[btac800-B24] Venkatesan A. et al (2017) SciLite: a platform for displaying text-mined annotations as a means to link research articles with biological data. Wellcome Open Res., 1, 25. 28948232 10.12688/wellcomeopenres.10210.2 PMC5527546

[btac800-B25] Wang L.L. et al (2020) CORD-19: the COVID-19 open research dataset. ArXiv [Preprint]. 2020 Apr 22:arXiv:2004.10706v4. PMID: 32510522; PMCID: PMC7251955.

